# Longitudinal relationships between glycemic status and body mass index in a multiethnic study: evidence from observational and genetic epidemiology

**DOI:** 10.1038/srep30744

**Published:** 2016-08-02

**Authors:** Adeola F. Ishola, Hertzel C. Gerstein, James C. Engert, Viswanathan Mohan, Rafael Diaz, Sonia S. Anand, David Meyre

**Affiliations:** 1 Department of Clinical Epidemiology and Biostatistics, McMaster University, Hamilton, Ontario, Canada; 2 Population Health Research Institute, McMaster University and Hamilton Health Sciences, Hamilton General Hospital, Hamilton, Ontario, Canada; 3 Department of Medicine, McMaster University, Hamilton, Ontario, Canada; 4 Research Institute of the McGill University Health Centre, Montreal, QC, Canada.; 5 Departments of Medicine and Human Genetics, McGill University, Montreal, QC, Canada; 6 Madras Diabetes Research Foundation, Chennai, India; 7 ECLA—Academic Research Organization, Rosario, Argentina; 8 Department of Pathology and Molecular Medicine, McMaster University, Hamilton, Ontario, Canada

## Abstract

We investigated the relationship between glycemic status and BMI and its interaction with obesity single-nucleotide polymorphisms (SNPs) in a multi-ethnic longitudinal cohort at high-risk for dysglycemia. We studied 17 394 participants from six ethnicities followed-up for 3.3 years. Twenty-three obesity SNPs were genotyped and an unweighted genotype risk score (GRS) was calculated. Glycemic status was defined using an oral glucose tolerance test. Linear regression models were adjusted for age, sex and population stratification. Normal glucose tolerance (NGT) to dysglycemia transition was associated with baseline BMI and BMI change. Impaired fasting glucose/impaired glucose tolerance to type 2 diabetes transition was associated with baseline BMI but not BMI change. No simultaneous significant main genetic effects and interactions between SNPs/GRS and glycemic status or transition on BMI level and BMI change were observed. Our data suggests that the interplay between glycemic status and BMI trajectory may be independent of the effects of obesity genes. This implies that individuals with different glycemic statuses may be combined together in genetic association studies on obesity traits, if appropriate adjustments for glycemic status are performed. Implementation of population-wide weight management programs may be more beneficial towards individuals with NGT than those at a later disease stage.

The worldwide obesity epidemic is mainly explained by environmental and lifestyle changes that occurred in the last five decades^1^. Increased consumption of high calorie food, lack of physical activity, and to a lesser extent sedentary behaviors, altered sleeping patterns, psychosocial stress, marital status are environmental factors predominantly attributed to driving the obesity epidemic^1^. However, there is a large inter-individual variability in body mass index (BMI) observed among populations exposed to an obesogenic environment, ranging from pathological leanness to morbid obesity^2^.

Heritability studies estimate that 47–90% of BMI variation is explained by genetic factors^3^. The inherited component of obesity has been further demonstrated by the discovery of genetic variants responsible for monogenic and polygenic forms of obesity^4^. Defects in eleven genes involved in the neuronal differentiation of the paraventricular nucleus and in the leptin/melanocortin pathway result in monogenic forms of obesity^5^. More recently, candidate gene, custom array-wide or genome-wide association studies (GWAS) have identified 130 loci convincingly associated with BMI or obesity^6^. Successful gene identification efforts led researchers to investigate whether gene variants may interact with specific environmental exposures to increase susceptibility to obesity^7^. For instance, single-nucleotide polymorphisms (SNPs) in *FTO* have been shown to interact with physical activity, dietary pattern or socio-economic status to increase BMI^8^,^9^,^10^,^11^.

In a systematic review, Chiu *et al*. found that in a majority of studies poor glucose regulation or insulin resistance, an important predictor of type 2 diabetes (T2D), was associated with weight loss in adults^12^. It was demonstrated that before diagnosis of T2D, weight tends to increase progressively overtime, whereas after diagnosis weight loss is common^12^. Furthermore, insulin resistance has also been shown to promote weight gain in adolescents^13^, but oppose further weight gain during adulthood^14^. The diverse findings suggests that the relationship between glycemic status and BMI maybe quite complex and influenced by multiple factors. Insulin exerts its effects on weight gain by inhibiting lipolysis, thus promoting fat storage^15^. On the other hand, weight loss may be attributed to progressive decline in pancreatic beta cell activity^16^. Furthermore, silent T2D patients tend to lose weight prior to being diagnosed because of the presence of glycosuria, a phenomenon that is amplified in health systems not able to routinely detect T2D at an early stage^17^. Intervention after T2D diagnosis also impacts weight trajectories. In particular, insulin or rosiglitazone medications promote weight gain^18^,^19^, whereas lifestyle intervention, glucagon-like peptide 1 agonists or amylin analogues promote weight loss^20^,^21^. To accurately determine the role of glycemic status in BMI evolution, before and after T2D diagnosis, more longitudinal studies are needed to clarify this complex relationship. Additionally, it is unclear whether the dynamic patterns of associations between BMI and T2D are influenced by biological factors, environmental exposures and their interactions. More specifically, understanding whether glycemic status may modify the BMI increasing effect of obesity predisposing SNPs has not been investigated to date.

Hence, we aimed to assess in 17 394 individuals from a multiethnic longitudinal study: (1) the association between baseline glycemic status and BMI level and BMI change during a 3.3 year follow-up; (2) the association between glycemic status transition and BMI level and BMI change; (3) the interaction between obesity predisposing SNPs and baseline glycemic status/glycemic status transition on BMI level and BMI change.

## Results

### Characteristics of the EpiDREAM cohort

The baseline clinical characteristics of participants are reported in Table 1. Of the 17 394 participants, 42.8%, 42.5% and 14.7% displayed normal glucose tolerance (NGT), impaired fasting glucose/impaired glucose tolerance (IFG/IGT) or T2D, respectively. The ethnic distribution of individuals was significantly different in the NGT, IFG/IGT and T2D groups. There was a decreasing percentage of IFG/IGT in subjects of East Asian (66.9%), African (62.8%), European (61.0%), Native North American (55.8%), Latino American (55.5%) and South Asian (43.5%) ancestry (Supplementary Table 3). There was a decreasing percentage of T2D in subjects of East Asian (22.7%), African (18.0%), South Asian (16.1%), European (14.4%), Latino American (12.9%) and Native North American (11.8%) ancestry (Supplementary Table 3). Overall, IFG/IGT and T2D subjects were on average 5.5–6.5 years older than the NGT subjects. The percentage of females was higher across all glycemic statuses (60.9%), but males were more likely to be IFG/IGT or T2D in comparison to NGT counterparts (Table 1).

### Effect of baseline glycemic status on BMI level and BMI change

IFG/IGT and T2D status were positively associated with BMI at baseline when compared with the NGT group (IFG/IGT: β = 0.34 ± 0.02, *P* = 1.35 × 10^−98^; T2D: β = 0.44 ± 0.02, *P* = 5.25 × 10^−80^; Table 2). There was an association between IFG/IGT status at baseline and lower BMI change during the 3.3 year follow-up in comparison with NGT subjects (IFG/IGT: β = −0.13 ± 0.03, *P* = 3.65 × 10^−7^). A significant negative association between T2D status at baseline and BMI change was also observed in comparison with NGT subjects (β = −0.34 ± 0.04, *P* = 1.06 × 10^−19^; Table 2). Fasting plasma glucose (FPG) and two-hour plasma glucose (2hPG) at baseline were positively associated with BMI at baseline (FPG: β = 0.19 ± 0.01, *P* = 1.80 × 10^−145^; 2hPG: β = 0.20 ± 0.01, *P* = 4.26 × 10^−159^; Table 2) and were negatively associated with BMI change during the follow-up period (FPG: β = −0.09 ± 0.01, *P* = 8.47 × 10^−14^ and 2hPG: β = −0.10 ± 0.01, *P* = 1.28 × 10^−19^; Table 2).

### Effect of glycemic status transition on BMI level and BMI change

The transition from NGT to IFG/IGT was positively associated with BMI at baseline (β = 0.19 ± 0.04, *P* = 2.60 × 10^−7^; Table 3) and BMI change (β = 0.19 ± 0.04, *P* = 2.93 × 10^−6^). Compared to stable NGT, converting from NGT to T2D showed a positive association with BMI at baseline (β = 0.36 ± 0.08, *P* = 4.35 × 10^−6^) and BMI change (β = 0.38 ± 0.09, *P* = 2.25 × 10^−5^), with two fold higher baseline and delta beta values compared to that observed in IFG/IGT converters. Similarly, the transition from IFG/IGT to T2D was positively associated with BMI at baseline (β = 0.20 ± 0.03, *P* = 6.28 × 10^−11^), in comparison to stable IFG/IGT over the study period. However, this association was not significant for BMI change (β = 0.06 ± 0.04, *P* = 8.28 × 10^−2^). The change in FPG levels was significantly associated with a positive increase in BMI change (β = 0.16 ± 0.01, *P* = 1.1 × 10^−52^), but was not associated with baseline BMI (β = −0.01 ± 0.01, *P* = 0.52). An association between change in 2hPG levels, with BMI at baseline (β = −0.03 ± 0.01, *P* = 1.6 ×10^−3^) as well as with BMI change (β = 0.17 ± 0.01, *P* = 2.4 × 10^−45^; Table 3) was also observed.

### Main association and interaction between obesity predisposing SNPs, glycemic status on BMI level and BMI change

Associations between the 23 obesity SNPs, the genotype risk score (GRS) and BMI at baseline, and BMI change are reported in Table 4. Three of 23 SNPs reached a significant association with BMI at baseline with a direction of effect consistent with published GWAS data: rs9939609 in *FTO*, rs2984618 in *TAL1,* and rs7903146 in *TCF7L2* (0.04 ≤ β ≤ 0.08; 1.4 × 10^−14^ ≤ *P* ≤ 1.8 × 10^−5^; Table 4). The GRS was also significantly associated with an increased BMI at baseline (β = 0.018 ± 0.002) per additional risk allele, *P* = 6.4 × 10^−14^, Table 4). The 23 SNPs and the GRS were not significantly associated with BMI change (Table 4).

Interactions between obesity SNPs/GRS and traits related to glycemic status on BMI at baseline and BMI change are reported in Table 4. There was only one significant interaction between the rs1011527 SNP in *LEPR* and FPG on baseline BMI (*P* = 3.9 × 10^−7^). However, no significant main effect was observed for the rs1011527 SNP in *LEPR* on BMI with (β = −0.02 ± 0.02; *P* = 0.22) or without (β = −0.01 ± 0.02; *P* = 0.72) the interaction term in the model. Interactions between obesity SNPs, GRS and traits related to glycemic status transition on BMI at baseline and BMI change are reported in Table 5. Overall, there was no simultaneous significant main effect and interaction between glycemic status traits or transition and obesity SNPs or GRS, in relation to BMI level and BMI change.

## Discussion

We sought to investigate the relationship between glycemic status and BMI evolution in the EpiDREAM study. Our results indicate that subjects with IFG/IGT or T2D at baseline have a higher BMI, compared to those with NGT, a result consistent with previous longitudinal studies in European populations^22^,^23^ but further extended to the multiethnic international EpiDREAM study. Since BMI level at baseline predicts worsened glucose homeostasis in our study, this demonstrates the important role of obesity as a driving factor for dysglycemia. Furthermore, IFG/IGT subjects gained less weight than NGT subjects during the 3.3 year follow-up period. Due to the role of insulin in fat storage^15^, the lower weight gain observed in individuals with prediabetes may be related to the progressive decline of their beta-cell function^16^. However, more experimental studies are needed to confirm this as the present study was not designed to examine this effect. In addition, the negative association between FPG, 2hPG and change in BMI is mainly explained by the weight loss experienced by T2D subjects (who display the higher baseline FPG and 2hPG values) during the follow-up. The weight loss observed in participants diagnosed with T2D at baseline may be attributed to glycosuria, an initial motivation to improve diet and lifestyle behaviors and medication for improvement of glycemia^24^. A consistent observation was made in a study by Feldstein and colleagues, where a subset of multiethnic participants with T2D from the USA lost weight within the first eighteen months after initial diagnosis^25^. However, by year three, they experienced a near complete weight regain, suggesting that sustained long term weight reduction is difficult to achieve for patients with T2D, especially in the presence of an “obesogenic” environment or weight increasing antidiabetic medication^25^,^26^.

Increased BMI at baseline and during the follow-up period is a strong driver for the transition from NGT to dysglycemia. This has been confirmed by other studies with participants of European ancestry which show a similar outcome, however our study extends the result to a multiethnic population^27^. Importantly, a higher BMI level at baseline but not BMI change is associated with IFG/IGT to T2D transition in our multiethnic population at risk for dysglycemia. Our finding provides support for the notion that obesity prevention may be a crucial factor in significantly decreasing the prevalence of dysglycemia worldwide. Conversely, this implies that in absence of intervention preexisting obesity plays a greater role than weight gain over time with respect to the IFG/IGT to T2D transition. Our findings suggest that preventative measures against further weight gain may be more beneficial towards individuals with NGT than those at a later disease stage. However, this is far from the reality of our current health care system, where weight loss is often proposed once individuals develop overt T2D. Collectively these findings support the notion of a strong interplay between BMI trajectories and evolution of glycemic status.

Despite the fact that subjects with NGT, IFG/IGT and T2D exhibit different BMI trajectories^28^, no simultaneous significant main effect and interaction between the obesity predisposing SNP/GRS and baseline glycemic status on BMI was observed. The interaction observed between the rs1011527 SNP in *LEPR* and FPG on baseline BMI is likely spurious. We propose that this spurious interaction results from the association between the rs1011527 SNP in *LEPR* and the interacting variable, FPG (β:0.05 ± 0.02; *P* = 1.9 × 10^−3^; data not shown). The variation in BMI attributed to obesity SNPs is similar for subjects with NGT, IFG/IGT and T2D, even though these groups display different patterns of BMI level and BMI change across time. These results have important implications in the design of genetic association studies for obesity traits. There is a persistent belief in the field of genetic epidemiology that subjects with T2D may be excluded from genetic association studies on obesity traits^29^. Likewise, the lack of interaction between glycemic status transition and obesity SNPs raises the possibility that the effect of obesity SNPs on BMI may be similar for those who maintain their glycemic status *versus* those who transition to a worsened state of glucose regulation. Consequently, if confirmed by future studies, our data may help to extend the types of designs where obesity genetic association studies can be performed (e.g. T2D case control design). We acknowledge the possibility of bias in the estimation of genetic effects when studies designed for a primary outcome (e.g. T2D in a T2D case control study) are investigated for secondary outcomes (e.g. BMI)^30^. However, these potential biases do not seem to represent a big obstacle to the discovery of novel obesity genes in practice, as illustrated by the initial discovery of the obesity gene *FTO* in a T2D case-control design^31^. Our data supports the view that individuals with different glycemic statuses can be pooled together in genetic association studies for obesity traits, if appropriate adjustments for glycemic status are performed.

The strength of this study lies in the quality of the phenotypic data. Measurements were performed with a standard protocol across the entire study. The oral glucose tolerance test (OGTT) is currently the gold standard method for epidemiological assessment glycemic status^32^. Additionally, the international longitudinal multiethnic design of EpiDREAM is ideal to assess the relationships between SNPs and BMI in interaction with the glycemic status. Likewise, the EpiDREAM cohort is enriched in subjects with newly diagnosed IFG/IGT or T2D, which optimizes the statistical power of gene x environment interaction tests in comparison to the general population in which these individuals are not as abundant. Nonetheless, we acknowledge that this particular design restricts the generalizability of our results. Other limitations include the non-exhaustive list of SNPs tested, the use of SNPs that may not represent the best proxies in non-European populations as they have been identified predominantly in European populations and the possibility that subtle true interaction effects may have been missed given the power of our study (Supplementary Figure 2). Other limitations are the small sample size of certain ethnic groups (e.g. East Asians). In addition, we did not have access to dietary and physical activity measurements at baseline and follow-up for the whole sample and were unable to adjust for these important covariates.

In summary, the interplay between glycemic status and BMI was confirmed at the epidemiological level, but no interaction between obesity predisposing SNPs and glycemic status was identified in this study. If confirmed by other studies and extended to more obesity predisposing SNPs, this finding will broaden the types of designs utilized for obesity genetic association studies. Our findings suggest that implementation of population-wide preventative measures against further weight gain may be more beneficial towards individuals with NGT than those at a later disease stage.

## Material and Methods

### Participants

The EpiDREAM study included 24872 individuals from 191 centres in 21 countries who were screened for eligibility to enter the DREAM clinical trial^33^. Individuals between the ages of 18–85 years, who were deemed to be at risk for dysglycemia defined by family history, ethnicity and abdominal obesity, were screened using a 75 gram OGTT from July 2001 to August 2, 2003. None of the study participants were taking glucose lowering medication at baseline, however a subset of the IFG/IGT patients enrolled in the DREAM clinical trial were given glucose lowering medication (rosiglitazone) during the follow-up. Detailed methods and description of the study cohort have been described elsewhere^33^,^34^. A total of 17394 subjects from six ethnic groups (East Asian, South Asian, European, African, Latin American and Native North American) and having both phenotypic and genotypic information available at baseline were included (Supplementary Figure 1). Of these 17394 individuals, 9297 have been prospectively followed for a median of 3.3 years (Supplementary Figure 1). Self-reported ethnicity has been validated in the 17394 individuals using the eigensoft software (http://genepath.med.harvard.edu/~reich/Software.htm). The EpiDREAM study was approved by local ethics committees. All experimental protocols were approved by McMaster University and were performed in accordance with relevant guidelines and regulations of McMaster University. Written informed consent was obtained from each subject prior to participation, in accordance with the Declaration of Helsinki.

### Genotyping

Buffy coats for DNA extraction were collected from all participants of the EpiDREAM study at baseline. DNA was extracted by the Gentra System. In total, 19 197 samples from the EpiDREAM genetic study were genotyped using the Illumina 50 K CVD Array (Supplementary Figure 1)^35^. We included 23 independent lead or proxy SNPs that reached genome-wide significant level of association (*P* < 5 × 10^−8^) for BMI and/or binary obesity status in literature and were available in the cardiovascular gene-centric 50 K SNP array (Supplementary Table 1). The 23 SNPs showed high genotyping call rates (99.96–100%; Supplementary Table 2). No deviation from Hardy-Weinberg equilibrium (HWE) was observed in the six ethnic groups (*P* ≥ 1 × 10^−6^).

### Phenotyping

Height (m) and weight (kg) were measured in clinical centers by a research staff. Standing height was measured to the nearest 0.1 cm with the participant looking straight ahead in bare feet and with his/her back against a wall. Weight was measured to the nearest 0.1 kg in light clothing. BMI was calculated as weight in kilograms (kg) divided by height in meters (m) squared. The BMI change value was calculated as the difference of the trait from baseline to follow-up.

We used the glycemic status information at baseline and follow-up. After an overnight fast of at least 8 hours, a fasting blood sample was collected from participants. After they consumed 75 g of anhydrous glucose in 300 ml of water, an additional blood sample was drawn after 2 hours. FPG and 2hPG levels were measured using an enzymatic reference method. The 2003 ADA criteria were used to classify participants as having normal glucose tolerance (NGT), as having impaired fasting glucose (IFG), as having impaired glucose tolerance (IGT), or as having T2D at baseline and at the end of the follow-up, as confirmed by an oral glucose tolerance test. NGT was defined as a fasting plasma glucose <5.6 mmol/L, IFG was defined as a fasting plasma glucose of 5.6 to 6.9 mmol/L, IGT was defined as a fasting plasma glucose below 7.0 mmol/L and a 2-h glucose between 7.8 and 11.0 mmol/L, and diabetes was defined if either the fasting plasma glucose was ≥7.0 mmol/L or the 2-h glucose was ≥11.1 mmol/L^36^.

### Statistical analyses

All statistical analyses were performed using SPSS 14.0 and R software. Genotypes were coded as 0, 1 and 2, depending on the number of copies of the risk alleles. The risk alleles were defined as alleles associated with increased BMI/risk of obesity in literature (Supplementary Table 2). All single SNP genetic association studies were performed under an additive model. A GRS was calculated by summing the alleles that increase BMI/obesity for the 23 SNPs. We used an unweighted score as previously recommended by Dudbridge^37^. Imputations were performed for the missing genotypes for each SNP in each ethnic group separately (n = 49 individuals; 0.012% of the total genotypes) using the arithmetic average of the coded genotypes observed for all the individuals successfully genotyped^38^.

Linear regression models were used to analyse the association between: (1) baseline glycemic status traits (IFG/IGT, T2D, dysglycemia, FPG and 2hPG) and BMI level and BMI change; (2) glycemic status transition traits (NGT to IFG/IGT, NGT to T2D, IFG/IGT to T2D, change in FPG and change in 2hPG) and BMI level and BMI change. Interactions between: (1) baseline glycemic status traits and 23 obesity predisposing SNPs on BMI level and BMI change and (2) glycemic status transition traits and 23 obesity predisposing SNPs on BMI level and BMI change were investigated by adding an interaction term to the regression model. For all analysis, our dependent variable was either baseline BMI or change in BMI. Our independent variables were either baseline glycemic status, glycemic status transition or 23 obesity predisposing SNPs, depending on the analysis being conducted. For the baseline glycemic status those with NGT were the reference group (coded as 0; 1 = IFG/IGT, T2D or dysglycemia). Values for BMI, FPG, 2hPG, change in BMI, change in FPG, and change in 2hPG were inverse normally transformed as they did not follow a normal distribution. All the tests performed were adjusted for age, sex, and population stratification/ethnicity. Additional adjustment for randomization to thiazolidinedione after baseline was implemented when the outcome was change in BMI.

Applying a Bonferroni corrected *P*-value across all the outcomes reduces the chance of making type I errors, but increases the chance of making type II errors. Therefore, we applied a separate Bonferroni correction to each research question^39^: (1) the association between baseline glycemic status and BMI, *P* < 6.3 × 10^−3^ (0.05/8), (2) the association between glycemic status transition and BMI, *P* < 5 × 10^−3^ (0.05/10) and the SNP/GRS by glycemic status interaction analyses, *P* < 4.2 × 10^−4^ (0.05/120).

## Additional Information

**How to cite this article**: Ishola, A. F. *et al*. Longitudinal relationships between glycemic status and body mass index in a multiethnic study: evidence from observational and genetic epidemiology. *Sci. Rep.* **6**, 30744; doi: 10.1038/srep30744 (2016).

## Supplementary Material

Supplementary Information

## Figures and Tables

**Table 1:** Baseline and follow-up characteristics of up to 17394 participants in EpiDREAM by glycemic status.

	Category	NGT	IFG/IGT	T2D	All	*P-* value
**Total at baseline N (%)**		7447 (42.8%)	7389 (42.5%)	2558 (14.7%)	17394 (100%)	
Gender N (%)	Male	2496 (33.5%)	3119 (42.2%)	1169 (45.7%)	6784 (39.1%)	1.34 × 10^−38^
	Female	4951 (66.5%)	4270 (57.8%)	1389 (54.3%)	10610 (60.9%)	
aAge (years)		49.38 (11.08)	54.90 (10.98)	55.79 (10.89)	52.66 (11.38)	1.44 × 10^−247^
aFPG (mmol/L)		4.85 (0.51) [7444]	5.86 (0.57) [7389]	7.54 (2.16) [2556]	5.67 (1.32) [17389]	0
a2hPG (mmol/L)		5.51 (1.19) [7419]	7.81 (1.83) [7388]	13.23 (3.95) [2424]	7.58 (3.26) [17231]	0
aBMI at baseline (kg/m2)		28.98 (6.04) [7437]	30.95 (6.12) [7380]	31.28 (6.34) [2556]	30.16 (6.20) [17373]	2.22 × 10^−104^
aBMI at follow-up (kg/m2)		29.34 (6.09) [3577]	31.42 (6.35) [4740]	31.05 (6.18) [980]	30.58 (6.31) [9297]	1.06 × 10^−50^
aBMI Change (kg/m2)		0.36 (2.83) [3555]	0.22 (2.54) [4683]	−0.52 (2.61) [971]	0.20 (2.67) [9209]	5.19 × 10^−19^
Ethnic groups N (%)						5.86 × 10^−76^
	South Asian	1556 (20.9%)	756 (10.2%)	444 (17.4%)	2756 (15.8%)	
	East Asian	74 (1.0%)	100 (1.4%)	51 (2.0%)	225 (1.3%)	
	European	3671 (49.3%)	4355 (58.9%)	1354 (52.9%)	9380 (53.9%)	
	African	465 (6.2%)	557 (7.5%)	225 (8.8%)	1247 (7.2%)	
	Latin American	1460 (19.6%)	1402 (19.0%)	425 (16.6%)	3287 (18.9%)	
	Native-North American	221 (3.0%)	219 (3.0%)	59 (2.3%)	499 (2.9%)	

NGT = Normal glucose tolerance; IFG = Impaired fasting glucose; IGT = Impaired glucose tolerance; T2D = Type 2 Diabetes; BMI = Body mass Index; FPG = fasting plasma glucose; 2hPG = two hour plasma glucose; SD = standard deviation; N = sample size.

^a^Data are presented as mean (SD) [N].

**Table 2:** Association between baseline glycemic traits and baseline BMI and change in BMI over 3.3 years.

	BMI	
	Baseline	Change
^a^IFG/IGT	0.34 ± 0.02(1.35 × 10^−98^)	−0.13 ± 0.03^b^(3.65 × 10^−7^)
^a^T2D	0.44 ± 0.02(5.25 × 10^−80^)	−0.34 ± 0.04^b^(1.06 × 10^−19^)
FPG	0.19 ± 0.01(1.80 × 10^−145^)	−0.09 ± 0.01^b^(8.47 × 10^−14^)
2hPG	0.20 ± 0.01(4.26 × 10^−159^)	−0.10 ± 0.01^b^(1.28 × 10^−19^)

NGT = Normal glucose tolerance; IFG = Impaired fasting glucose; IGT = Impaired glucose tolerance; T2D = Type 2 Diabetes; BMI = Body mass Index; FPG = fasting plasma glucose; 2hPG = two hour plasma glucose. *P* value < 6.3 × 10^−3^ (0.05/8) are statistically significant; data are presented as B ± SE (*P*-value). ^a^NGT used as the reference group. Adjusted for sex, age and population stratification/ethnicity and ^b^randomization to thiazolidinedione after baseline. Linear regression analysis was performed.

**Table 3:** Association between glycemic transition traits and baseline BMI and change in BMI over 3.3 years.

Glycemic status transition	BMI	
	Baseline	Change
^a^NGT to IFG/IGT	0.19 ± 0.04(2.60 × 10^−7^)	0.19 ± 0.04(2.93 × 10^−6^)
^a^NGT to T2D	0.36 ± 0.08(4.35 × 10^−6^)	0.38 ± 0.09(2.25 × 10^−5^)
^b^IFG/IGT to T2D	0.20 ± 0.03(6.28 × 10^−11^)	0.06 ± 0.04^c^(8.28 × 10^−2^)
Change in FPG	−0.01 ± 0.01 (0.52)	0.16 ± 0.01^c^(1.1 × 10^−52^)
Change in 2hPG	−0.03 ± 0.01(1.6 × 10^−3^)	0.17 ± 0.01^c^(2.4 × 10^−45^)

NGT = Normal glucose tolerance; IFG = Impaired fasting glucose; IGT = Impaired glucose tolerance; T2D = Type 2 Diabetes; BMI = Body mass Index. ^a^Reference group = NGT subjects who remain NGT at follow-up; ^b^Reference group = IFG/IGT subjects who remain IFG/IGT at baseline. N_NGT to IFG/IGT_ = 972; n_NGT to T2D_ = 167; n_IFG/IGT to T2D_ = 1104. Linear regression analysis was performed.

**Table 4:** Interactions between obesity SNPs or GRS and glycemic traits on BMI at baseline and change in BMI.

Gene	SNP	Risk Allele	SNP Main effect^b^	IFG/IGT Interaction	Dysglycemia Interaction	T2D Interaction	FPG Interaction	2hPG Interaction
BMI AT BASELINE								
*APOE*	rs2075650	G	0.01 ± 0.02 (0.15)	0.03 ± 0.03 (0.32)	0.02 ± 0.03 (0.44)	0.002 ± 0.050 (0.96)	−0.01 ± 0.01 (0.50)	0.004 ± 0.015 (0.81)
*BDNF*	rs6265	C	0.02 ± 0.01 (0.19)	0.04 ± 0.03 (0.18)	0.03 ± 0.03 (0.22)	0.01 ± 0.04 (0.77)	0.01 ± 0.01 (0.34)	0.01 ± 0.01 (0.67)
*BDNF*	rs1401635	G	0.02 ± 0.01 (0.11)	0.03 ± 0.02 (0.29)	0.01 ± 0.02 (0.80)	−0.05 ± 0.03 (0.15)	−0.02 ± 0.01 (0.16)	−0.01 ± 0.01 (0.18)
*CDKAL1*	rs2206734	C	0.04 ± 0.01 (3.9 × 10^−3^)	0.004 ± 0.027 (0.87)	0.01 ± 0.02 (0.55)	0.05 ± 0.04 (0.17)	0.03 ± 0.01 (3.7 × 10^−2^)	0.03 ± 0.01 (7.2 × 10^−3^)
*FANCL*	rs12617233	C	0.01 ± 0.01 (0.28)	−0.02 ± 0.02 (0.40)	−0.01 ± 0.02 (0.47)	−0.001 ± 0.031 (0.97)	−0.01 ± 0.01 (0.16)	−0.002 ± 0.010 (0.84)
*FTO*	rs7203521	A	0.01 ± 0.01 (0.48)	−0.01 ± 0.02 (0.75)	−0.01 ± 0.02 (0.78)	−0.005 ± 0.030 (0.87)	0.002 ± 0.010 (0.82)	−0.01 ± 0.01 (0.60)
*FTO*	rs9939609	A	**0.08** ± **0.01 (1.4** × **10**^**−14**^)	−0.02 ± 0.02 (0.40)	−0.02 ± 0.02 (0.43)	−0.01 ± 0.03 (0.74)	−0.02 ± 0.01 (1.5 × 10^−2^)	−0.01 ± 0.01 (0.31)
*GIPR*	rs11671664	G	0.03 ± 0.02 (0.11)	0.04 ± 0.03 (0.29)	0.05 ± 0.03 (0.15)	0.08 ± 0.05 (0.10)	0.03 ± 0.02 (9.0 × 10^−2^)	0.01 ± 0.02 (0.25)
*ITIH4*	rs2535633	G	−0.002 ± 0.015 (0.13)	−0.005 ± 0.022 (0.82)	−0.01 ± 0.02 (0.78)	−0.01 ± 0.03 (0.66)	0.01 ± 0.01 (0.36)	0.0004 ± 0.009 (0.96)
*KAT8*	rs749767	A	0.01 ± 0.01 (0.39)	0.03 ± 0.02 (0.13)	0.02 ± 0.02 (0.32)	−0.01 ± 0.03 (0.69)	−0.01 ± 0.01 (0.17)	−0.002 ± 0.010 (0.80)
*LEPR*	rs1011527	A	−0.01 ± 0.02 (0.72)	−0.04 ± 0.03 (0.20)	−0.05 ± 0.03 (9.7 × 10^−2^)	−0.08 ± 0.05 (8.2 × 10^−2^)	**−0.07** ± **0.01 (3.9** × **10**^**−7**^)	−0.02 ± 0.01 (0.23)
*MAPK25*	rs997295	T	0.01 ± 0.02 (0.41)	−0.02 ± 0.02 (0.34)	−0.01 ± 0.02 (0.55)	0.01 ± 0.03 (0.64)	0.02 ± 0.01 (8.1 × 10^−2^)	0.004 ± 0.010 (0.66)
*NPC1*	rs1805081	A	0.02 ± 0.01 (0.15)	0.03 ± 0.02 (0.26)	0.02 ± 0.02 (0.35)	0.002 ± 0.033 (0.95)	−0.01 ± 0.01 (0.20)	−0.01 ± 0.01 (0.53)
*NT5C2*	rs3824755	C	0.03 ± 0.01 (3.5 × 10^−2^)	−0.02 ± 0.03 (0.54)	−0.03 ± 0.03 (0.24)	−0.08 ± 0.04 (7.6 × 10^−2^)	−0.04 ± 0.01 (6.5 × 10^−3^)	−0.02 ± 0.01 (0.20)
*NTRK2*	rs1211166	A	0.01 ± 0.01 (0.28)	−0.02 ± 0.03 (0.43)	−0.02 ± 0.02 (0.43)	−0.02 ± 0.03 (0.62)	−0.002 ± 0.012 (0.86)	−0.02 ± 0.01 (0.16)
*PCSK1*	rs6232	C	0.02 ± 0.03 (0.32)	−0.02 ± 0.05 (0.67)	−0.02 ± 0.05 (0.72)	−0.001 ± 0.075 (0.99)	−0.02 ± 0.02 (0.29)	−0.02 ± 0.02 (0.48)
*PCSK1*	rs6235	G	0.002 ± 0.012 (0.85)	−0.04 ± 0.02 (0.15)	−0.04 ± 0.02 (0.11)	−0.03 ± 0.04 (0.33)	−0.02 ± 0.01 (2.9 × 10^−2^)	−0.01 ± 0.01 (0.55)
*POMC*	rs7605927	G	−0.01 ± 0.01 (0.24)	−0.01 ± 0.02 (0.64)	−0.01 ± 0.02 (0.73)	−0.001 ± 0.031 (0.99)	−0.03 ± 0.01 (7.7 × 10^−3^)	−0.01 ± 0.01 (0.35)
*TAL1*	rs2984618	T	**0.04** ± **0.01 (1.8** × **10**^**−5**^)	−0.01 ± 0.02 (0.77)	−0.01 ± 0.02 (0.50)	−0.04 ± 0.03 (0.15)	−0.02 ± 0.01 (6.3 × 10^−2^)	−0.01 ± 0.01 (0.47)
*TCF7L2*	rs7903146	C	**0.05** ± **0.01 (1.7** × **10**^**−6**^)	0.03 ± 0.02 (0.24)	0.02 ± 0.02 (0.39)	0.005 ± 0.033 (0.89)	0.01 ± 0.01 (0.27)	0.02 ± 0.01 (2.5 × 10^−2^)
*TFAP2B*	rs2272903	G	0.02 ± 0.01 (0.13)	0.04 ± 0.03 (0.21)	0.03 ± 0.03 (0.31)	0.01 ± 0.04 (0.90)	0.02 ± 0.01 (0.11)	0.003 ± 0.014 (0.81)
*TNN13K*	rs1514176	G	0.03 ± 0.01 (5.6 × 10^−3^)	0.002 ± 0.021 (0.91)	−0.004 ± 0.020 (0.85)	−0.02 ± 0.03 (0.42)	−0.02 ± 0.01 (7.0 × 10^−2^)	−0.004 ± 0.098 (0.67)
*USP37*	rs611203	G	−0.002 ± 0.010 (0.83)	0.04 ± 0.02 (0.10)	0.04 ± 0.02 (4.3 × 10^−2^)	0.06 ± 0.03 (6.6 × 10^−2^)	0.02 ± 0.01 (9.6 × 10^−2^)	0.01 ± 0.01 (0.57)
**Genotype score**			**0.018** ± **0.002 (6.4** × **10**^**−14**^)	0.003 ± 0.005 (0.58)	0.0004 ± 0.0050 (0.92)	−0.01 ± 0.01 (0.40)	−0.006 ± 0.002 (6.3 × 10^−3^)	−0.002 ± 0.002 (0.47)
CHANGE IN BMI^a^								
*APOE*	rs2075650	G	−0.01 ± 0.02 (0.56)	**−**0.04 ± 0.05 (0.43)	−0.003 ± 0.045 (0.93)	0.13 ± 0.08 (8.4 × 10^−2^)	0.01 ± 0.02 (0.70)	−0.01 ± 0.02 (0.82)
*BDNF*	rs6265	C	0.01 ± 0.02 (0.48)	−0.07 ± 0.04 (7.1 × 10^−2^)	−0.07 ± 0.04 (8.1 × 10^−2^)	−0.04 ± 0.07 (0.55)	**−**0.03 ± 0.02 (0.15)	−0.01 ± 0.02 (0.49)
*BDNF*	rs1401635	G	0.02 ± 0.02 (0.16)	0.02 ± 0.03 (0.47)	0.03 ± 0.03 (0.40)	0.05 ± 0.06 (0.35)	0.02 ± 0.02 (0.31)	0.02 ± 0.02 (0.15)
*CDKAL1*	rs2206734	C	−0.05 ± 0.02 (3.4 × 10^−3^)	0.04 ± 0.04 (0.26)	0.05 ± 0.04 (0.22)	0.04 ± 0.06(0.50)	−0.002 ± 0.019 (0.93)	0.01 ± 0.02 (0.71)
*FANCL*	rs12617233	C	−0.03 ± 0.01 (2.8 × 10^−2^)	0.05 ± 0.03 (0.12)	0.05 ± 0.03 (0.12)	0.03 ± 0.05 (0.59)	0.01 ± 0.02 (0.43)	0.03 ± 0.02 (3.2 × 10^−2^)
*FTO*	rs7203521	A	0.01 ± 0.01 (0.64)	0.06 ± 0.03 (5.5 × 10^−2^)	0.06 ± 0.03 (4.1 × 10^−2^)	0.06 ± 0.05 (0.20)	0.01 ± 0.02 (0.97)	0.01 ± 0.02 (0.58)
*FTO*	rs9939609	A	−0.0001 ± 0.015 (0.99)	0.07 ± 0.03 (2.5 × 10^−2^)	0.07 ± 0.03 (2.4 × 10^−2^)	0.05 ± 0.05 (0.37)	0.03 ± 0.02 (5.3 × 10^−2^)	0.02 ± 0.02 (0.26)
*GIPR*	rs11671664	G	−0.001 ± 0.024 (0.95)	−0.05 ± 0.05 (0.28)	−0.06 ± 0.05 (0.25)	−0.06 ± 0.08 (0.45)	0.001 ± 0.025 (0.96)	−0.02 ± 0.02 (0.44)
*ITIH4*	rs2535633	G	0.003 ± 0.015 (0.83)	−0.04 ± 0.03 (0.21)	−0.02 ± 0.03 (0.49)	0.06 ± 0.05 (0.22)	0.01 ± 0.02 (0.58)	0.001 ± 0.015 (0.97)
*KAT8*	rs749767	A	0.01 ± 0.02 (0.35)	−0.05 ± 0.03 (0.10)	−0.03 ± 0.03 (0.30)	0.07 ± 0.05 (0.16)	−0.02 ± 0.02 (0.20)	0.02 ± 0.02 (0.22)
*LEPR*	rs1011527	A	−0.02 ± 0.02 (0.41)	0.02 ± 0.05 (0.62)	0.04 ± 0.05 (0.38)	0.13 ± 0.08 (9.9 × 10^−2^)	0.03 ± 0.02 (0.20)	0.04 ± 0.02 (0.13)
*MAPK25*	rs997295	T	0.002 ± 0.015 (0.99)	0.02 ± 0.03 (0.54)	0.01 ± 0.03 (0.70)	−0.02 ± 0.05 (0.63)	0.002 ± 0.015 (0.89)	−0.01 ± 0.02 (0.44)
*NPC1*	rs1805081	A	0.003 ± 0.016 (0.83)	−0.02 ± 0.03 (0.49)	−0.03 ± 0.03 (0.39)	−0.04 ± 0.05 (0.48)	−0.03 ± 0.02 (4.2 × 10^−2^)	-0.02 ± 0.02 (0.33)
*NT5C2*	rs3824755	C	0.02 ± 0.02 (0.31)	−0.08 ± 0.04 (8.1 × 10^−2^)	−0.09 ± 0.04 (3.8 × 10^−2^)	−0.14 ± 0.07 (5.3 × 10^−2^)	−0.03 ± 0.02 (0.19)	−0.03 ± 0.02 (0.22)
*NTRK2*	rs1211166	A	−0.002 ± 0.018 (0.92)	−0.03 ± 0.04 (0.43)	−0.02 ± 0.04 (0.49)	0.01 ± 0.06 (0.83)	−0.03 ± 0.02 (0.12)	−0.02 ± 0.02 (0.39)
*PCSK1*	rs6232	C	−0.03 ± 0.04 (0.49)	0.21 ± 0.08 (6.0 × 10^−3^)	0.17 ± 0.08 (2.2 × 10^−2^)	−0.01 ± 0.12 (0.95)	0.07 ± 0.04 (8.5 × 10^−2^)	0.02 ± 0.04 (0.63)
*PCSK1*	rs6235	G	−0.002 ± 0.017 (0.93)	0.02 ± 0.04 (0.49)	0.005 ± 0.034 (0.89)	−0.08 ± 0.06 (0.15)	0.02 ± 0.02 (0.21)	0.001 ± 0.018 (0.92)
*POMC*	rs7605927	G	−0.01 ± 0.02 (0.51)	−0.01 ± 0.03 (0.68)	−0.01 ± 0.03 (0.74)	0.02 ± 0.05 (0.74)	−0.0004 ± 0.0157 (0.98)	−0.01 ± 0.02 (0.66)
*TAL1*	rs2984618	T	−0.01 ± 0.01 (0.38)	−0.03 ± 0.03 (0.25)	−0.04 ± 0.03 (0.19)	−0.03 ± 0.05 (0.54)	−0.02 ± 0.01 (0.31)	−0.01 ± 0.01 (0.43)
*TCF7L2*	rs7903146	C	0.02 ± 0.02 (0.20)	0.02 ± 0.03 (0.63)	0.01 ± 0.03 (0.76)	−0.04 ± 0.06 (0.43)	0.01 ± 0.02 (0.72)	0.003 ± 0.017 (0.86)
*TFAP2B*	rs2272903	G	−0.03 ± 0.02 (0.13)	−0.04 ± 0.04 (0.36)	−0.03 ± 0.04 (0.45)	0.002 ± 0.072 (0.97)	0.002 ± 0.021 (0.92)	0.01 ± 0.02 (0.68)
*TNN13K*	rs1514176	G	−0.02 ± 0.01 (0.29)	−0.06 ± 0.03 (6.4 × 10^−2^)	−0.05 ± 0.03 (8.8 × 10^−2^)	−0.03 ± 0.05 (0.62)	−0.03 ± 0.02 (7.5 × 10^−2^)	−0.03 ± 0.01 (8.5 × 10^−2^)
*USP37*	rs611203	G	−0.02 ± 0.01 (0.24)	−0.06 ± 0.03 (7.6 × 10^−2^)	−0.06 ± 0.03 (5.7 × 10^−2^)	−0.06 ± 0.05 (0.22)	−0.03 ± 0.02 (4.8 × 10^−2^)	−0.02 ± 0.02 (0.21)
**Genotype score**			−0.004 ± 0.003 (0.24)	−0.01 ± 0.01 (0.41)	−0.004 ± 0.007 (0.53)	0.004 ± 0.012 (0.72)	−0.003 ± 0.004 (0.36)	−0.0003 ± 0.0035 (0.92)

Data are indicated as B ± SE (*P*-value). Adjusted for sex, age and population stratification/ethnicity and ^a^randomization to thiazolidinedione after baseline. *P* value < 4.2 × 10^−4^ (0.05/120) are statistically significant interactions. ^b^*P* value <* *2.1 × 10^−3^ (0.05/24) are statistically significant main effects (without interaction term in model). Genotype score was calculated by summing the alleles predisposing to increased BMI/obesity for the 23 SNPs.

**Table 5:** Interaction between obesity SNPs or GRS and glycemic transition on baseline BMI and change in BMI.

Gene	SNP	Risk Allele	SNP Main effect^b^	NGT to IFG/IGT Interaction	NGT to T2D Interaction	IFG/IGT to T2D Interaction	Change in FPG Interaction	Change in 2hPG Interaction
BMI AT BASELINE								
*APOE*	rs2075650	G	0.01 ± 0.02 (0.15)	0.003 ± 0.081 (0.97)	0.06 ± 0.15 (0.69)	−0.09 ± 0.06 (0.14)	0.0002 ± 0.0194 (0.99)	−0.01 ± 0.02 (0.54)
*BDNF*	rs6265	C	0.02 ± 0.01 (0.19)	−0.04 ± 0.07 (0.53)	0.06 ± 0.16 (0.68)	−0.07 ± 0.05 (0.21)	−0.01 ± 0.02 (0.51)	0.005 ± 0.019 (0.81)
*BDNF*	rs1401635	G	0.02 ± 0.01 (0.11)	−0.10 ± 0.06 (8.3 × 10^−2^)	−0.02 ± 0.12 (0.84)	−0.05 ± 0.05 (0.30)	−0.004 ± 0.014 (0.79)	0.001 ± 0.016 (0.94)
*CDKAL1*	rs2206734	C	0.04 ± 0.01 (3.9 × 10^−3^)	–0.02 ± 0.07 (0.72)	−0.12 ± 0.14 (0.37)	−0.03 ± 0.05 (0.57)	−0.03 ± 0.02 (4.8 × 10^−2^)	0.001 ± 0.018 (0.93)
*FANCL*	rs12617233	C	0.01 ± 0.01 (0.28)	0.01 ± 0.05 (0.80)	−0.01 ± 0.11 (0.91)	−0.07 ± 0.04 (0.10)	−0.01 ± 0.01 (0.29)	0.01 ± 0.01 (0.50)
*FTO*	rs7203521	A	0.01 ± 0.01 (0.48)	0.04 ± 0.05 (0.38)	0.05 ± 0.10 (0.62)	0.05 ± 0.04 (0.27)	0.01 ± 0.01 (0.39)	−0.01 ± 0.01 (0.58)
*FTO*	rs9939609	A	**0.08** ± **0.01 (1.4** × **10**^**−14**^)	0.04 ± 0.05 (0.44)	−0.01 ± 0.11 (0.94)	0.06 ± 0.04 (0.15)	0.03 ± 0.01 (4.8 × 10^−2^)	−0.001 ± 0.015 (0.97)
*GIPR*	rs11671664	G	0.03 ± 0.02 (0.11)	0.05 ± 0.09 (0.57)	−0.03 ± 0.18 (0.85)	−0.02 ± 0.06 (0.78)	−0.01 ± 0.02 (0.54)	−0.005 ± 0.023 (0.84)
*ITIH4*	rs2535633	G	−0.002 ± 0.015 (0.13)	−0.004 ± 0.052 (0.94)	−0.07 ± 0.11 (0.52)	−0.0004 ± 0.0427 (0.99)	−0.01 ± 0.01 (0.52)	0.01 ± 0.01 (0.53)
*KAT8*	rs749767	A	0.01 ± 0.01 (0.39)	0.01 ± 0.05 (0.80)	0.04 ± 0.11 (0.75)	0.05 ± 0.04 (0.25)	0.002 ± 0.013 (0.89)	−0.01 ± 0.01 (0.34)
*LEPR*	rs1011527	A	−0.01 ± 0.02 (0.72)	−0.10 ± 0.08 (0.17)	−0.30 ± 0.16 (6.4 × 10^−2^)	−0.14 ± 0.06 (3.3 × 10^−2^)	0.03 ± 0.02 (0.11)	−0.01 ± 0.02 (0.74)
*MAPK25*	rs997295	T	0.01 ± 0.02 (0.41)	0.02 ± 0.05 (0.73)	0.15 ± 0.11 (0.17)	−0.04 ± 0.04 (0.38)	0.01 ± 0.01 (0.62)	−0.01 ± 0.01 (0.68)
*NPC1*	rs1805081	A	0.02 ± 0.01 (0.15)	−0.02 ± 0.05 (0.72)	−0.09 ± 0.12 (0.44)	0.01 ± 0.04 (0.87)	0.004 ± 0.014 (0.78)	−0.01 ± 0.02 (0.46)
*NT5C2*	rs3824755	C	0.03 ± 0.01 (3.5 × 10^−2^)	−0.11 ± 0.07 (0.12)	−0.23 ± 0.14 (9.7 × 10^−2^)	−0.01 ± 0.06 (0.88)	0.01 ± 0.02 (0.71)	−0.03 ± 0.02 (0.11)
*NTRK2*	rs1211166	A	0.01 ± 0.01 (0.28)	−0.11 ± 0.06 (8.2 × 10^−2^)	0.11 ± 0.14 (0.43)	0.03 ± 0.05 (0.05)	0.01 ± 0.02 (0.58)	0.02 ± 0.02 (0.16)
*PCSK1*	rs6232	C	0.02 ± 0.03 (0.32)	0.01 ± 0.13 (0.93)	−0.36 ± 0.29 (0.21)	−0.13 ± 0.10 (0.20)	0.04 ± 0.03 (0.25)	−0.03 ± 0.03 (0.44)
*PCSK1*	rs6235	G	0.002 ± 0.012 (0.85)	0.06 ± 0.06 (0.27)	−0.12 ± 0.12 (0.31)	0.04 ± 0.05 (0.44)	0.004 ± 0.015 (0.78)	0.001 ± 0.017 (0.97)
*POMC*	rs7605927	**G**	−0.01 ± 0.01 (0.24)	−0.03 ± 0.05 (0.52)	−0.03 ± 0.12 (0.77)	0.004 ± 0.044 (0.93)	0.02 ± 0.01 (0.17)	−0.0004 ± 0.0153 (0.98)
*TAL1*	rs2984618	T	**0.04** ± **0.01 (1.8** × **10**^**−5**^)	−0.05 ± 0.05 (0.28)	−0.11 ± 0.11 (0.32)	−0.04 ± 0.04 (0.38)	−0.004 ± 0.013 (0.76)	−0.01 ± 0.01 (0.36)
*TCF7L2*	rs7903146	C	**0.05** ± **0.01 (1.7** × **10**^**−6**^)	0.04 ± 0.06 (0.47)	−0.04 ± 0.11 (0.70)	0.01 ± 0.04 (0.75)	−0.01 ± 0.01 (0.60)	−0.02 ± 0.02 (0.16)
*TFAP2B*	rs2272903	G	0.02 ± 0.01 (0.13)	0.09 ± 0.07 (0.19)	0.11 ± 0.14 (0.46)	0.08 ± 0.06 (0.20)	0.004 ± 0.018 (0.83)	−0.005 ± 0.021 (0.83)
*TNN13K*	rs1514176	G	0.03 ± 0.01 (5.6 × 10^−3^)	0.01 ± 0.05 (0.85)	−0.23 ± 0.11 (3.0 × 10^−2^)	0.001 ± 0.042 (0.99)	0.01 ± 0.01 (0.28)	−0.01 ± 0.01 (0.49)
*USP37*	rs611203	G	−0.002 ± 0.010 (0.83)	0.01 ± 0.05 (0.87)	0.06 ± 0.11 (0.56)	0.01 ± 0.04 (0.78)	0.003 ± 0.014 (0.85)	0.002 ± 0.014 (0.85)
**Genotype score**			**0.018** ± **0.002 (6.4** × **10**^**−14**^)	−0.004 ± 0.012 (0.73)	−0.03 ± 0.03 (0.19)	−0.004 ± 0.009 (0.69)	0.003 ± 0.003 (0.35)	−0.004 ± 0.003 (0.26)
CHANGE IN BMI^a^								
*APOE*	rs2075650	G	−0.01 ± 0.02 (0.56)	0.07 ± 0.09 (0.42)	−0.06 ± 0.18 (0.73)	0.13 ± 0.07 (8.9 × 10^−2^)	0.05 ± 0.02 (3.3 × 10^−2^)	0.03 ± 0.02 (0.19)
*BDNF*	rs6265	C	0.01 ± 0.02 (0.48)	−0.08 ± 0.07 (0.27)	−0.24 ± 0.17 (0.16)	0.13 ± 0.06 (3.4 × 10^−2^)	0.03 ± 0.01 (7.8 × 10^−2^)	0.04 ± 0.02 (7.2 × 10^−2^)
*BDNF*	rs1401635	G	0.02 ± 0.02 (0.16)	−0.09 ± 0.06 (0.16)	−0.02 ± 0.14 (0.89)	−0.01 ± 0.05 (0.87)	−0.01 ± 0.02 (0.43)	0.02 ± 0.02 (0.15)
*CDKAL1*	rs2206734	C	−0.05 ± 0.02 (3.4 × 10^−3^)	−0.01 ± 0.07 (0.92)	−0.13 ± 0.16 (0.43)	0.02 ± 0.06 (0.73)	−0.02 ± 0.02 (0.34)	0.0002 ± 0.0193 (0.99)
*FANCL*	rs12617233	C	−0.03 ± 0.01 (2.8 × 10^−2^)	−0.03 ± 0.06 (0.55)	0.10 ± 0.12 (0.42)	−0.04 ± 0.05 (0.39)	−0.01 ± 0.02 (0.70)	−0.03 ± 0.02 (8.9 × 10^−2^)
*FTO*	rs7203521	A	0.01 ± 0.01 (0.64)	0.03 ± 0.05 (0.53)	0.07 ± 0.12 (0.59)	0.09 ± 0.05 (7.4 × 10^−2^)	0.01 ± 0.02 (0.41)	0.01 ± 0.02 (0.46)
*FTO*	rs9939609	A	−0.0001 ± 0.0150 (0.99)	−0.02 ± 0.06 (0.70)	−0.10 ± 0.12 (0.42)	−0.06 ± 0.05 (0.20)	−0.02 ± 0.01 (0.20)	−0.03 ± 0.02 (0.11)
*GIPR*	rs11671664	G	−0.001 ± 0.024 (0.95)	0.07 ± 0.09 (0.46)	−0.30 ± 0.21 (0.15)	0.02 ± 0.08 (0.82)	0.01 ± 0.02 (0.54)	−0.01 ± 0.03 (0.58)
*ITIH4*	rs2535633	G	0.003 ± 0.015 (0.83)	0.09 ± 0.06 (9.5 × 10^−2^)	0.22 ± 0.12 (6.9 × 10^−2^)	−0.04 ± 0.05 (0.40)	0.01 ± 0.01 (0.33)	0.03 ± 0.02 (3.4 × 10^−2^)
*KAT8*	rs749767	A	0.01 ± 0.02 (0.35)	−0.05 ± 0.06 (0.34)	−0.22 ± 0.13 (0.10)	−0.05 ± 0.05 (0.36)	−0.01 ± 0.01 (0.45)	0.002 ± 0.016 (0.92)
*LEPR*	rs1011527	A	−0.02 ± 0.02 (0.41)	−0.14 ± 0.09 (0.11)	0.03 ± 0.18 (0.85)	0.05 ± 0.08 (0.52)	−0.03 ± 0.02 (0.21)	−0.02 ± 0.03 (0.51)
*MAPK25*	rs997295	T	0.002 ± 0.015 (0.99)	0.05 ± 0.05 (0.35)	0.09 ± 0.12 (0.46)	−0.02 ± 0.05 (0.75)	0.02 ± 0.01 (0.15)	0.02 ± 0.02 (0.26)
*NPC1*	rs1805081	A	0.003 ± 0.016 (0.83)	−0.04 ± 0.06 (0.44)	0.08 ± 0.14 (0.58)	−0.08 ± 0.05 (0.13)	−0.001 ± 0.016 (0.96)	−0.01 ± 0.02 (0.40)
*NT5C2*	rs3824755	C	0.02 ± 0.02 (0.31)	−0.07 ± 0.08 (0.36)	−0.07 ± 0.16 (0.64)	0.01 ± 0.07 (0.85)	−0.02 ± 0.02 (0.44)	0.01 ± 0.02 (0.73)
*NTRK2*	rs1211166	A	−0.002 ± 0.018 (0.92)	−0.03 ± 0.07 (0.68)	0.07 ± 0.16 (0.65)	−0.04 ± 0.06 (0.48)	0.03 ± 0.02 (9.3 × 10^−2^)	−0.02 ± 0.02 (0.38)
*PCSK1*	rs6232	C	−0.03 ± 0.04 (0.49)	0.28 ± 0.14 (4.9 × 10^−2^)	0.44 ± 0.31 (0.15)	−0.07 ± 0.12 (0.57)	−0.06 ± 0.04 (0.12)	−0.02 ± 0.04 (0.64)
*PCSK1*	rs6235	G	−0.002 ± 0.017 (0.93)	0.07 ± 0.06 (0.24)	−0.04 ± 0.13 (0.77)	−0.06 ± 0.06 (0.26)	−0.003 ± 0.017 (0.86)	−0.01 ± 0.02 (0.51)
*POMC*	rs7605927	G	−0.01 ± 0.02 (0.51)	0.02 ± 0.06 (0.75)	0.05 ± 0.13 (0.70)	0.01 ± 0.05 (0.87)	−0.01 ± 0.02 (0.57)	0.001 ± 0.017 (0.93)
*TAL1*	rs2984618	T	−0.01 ± 0.01 (0.38)	0.05 ± 0.05 (0.32)	0.05 ± 0.13 (0.70)	−0.04 ± 0.05 (0.36)	−0.02 ± 0.01 (0.20)	0.01 ± 0.02 (0.35)
*TCF7L2*	rs7903146	C	0.02 ± 0.02 (0.20)	−0.003 ± 0.062 (0.97)	0.19 ± 0.13 (0.15)	−0.06 ± 0.05 (0.21)	−0.01 ± 0.02 (0.35)	0.001 ± 0.017 (0.97)
*TFAP2B*	rs2272903	G	−0.03 ± 0.02 (0.13)	0.11 ± 0.08 (0.17)	−0.004 ± 0.160 (0.98)	0.09 ± 0.07 (0.22)	0.02 ± 0.02 (0.29)	−0.002 ± 0.023 (0.92)
*TNN13K*	rs1514176	G	−0.02 ± 0.01 (0.29)	0.10 ± 0.05 (6.0 × 10^−2^)	0.15 ± 0.12 (0.22)	−0.03 ± 0.05 (0.55)	0.02 ± 0.01 (0.14)	0.01 ± 0.02 (0.53)
*USP37*	rs611203	G	−0.02 ± 0.01 (0.24)	0.04 ± 0.06 (0.50)	−0.12 ± 0.12 (0.33)	0.01 ± 0.05 (0.89)	0.02 ± 0.01 (0.19)	−0.002 ± 0.016 (0.88)
**Genotype score**			−0.004 ± 0.003 (0.24)	0.01 ± 0.01 (0.37)	0.01 ± 0.03 (0.65)	−0.01 ± 0.01 (0.41)	0.003 ± 0.003 (0.34)	0.002 ± 0.004 (0.49)

Data are indicated as B ± SE (*P*). Adjusted for sex, age, population stratification/ethnicity and ^a^randomization to thiazolidinedione after baseline. *P* < 4.1 × 10^−4^ (0.05/120) are statistically significant interactions. *P* < 2.1 × 10^−3^ (0.05/24) are statistically significant main effects (without interaction term in model)^b^. Genotype score was calculated by summing the alleles predisposing to increased BMI/obesity for the 23 SNPs.
