# Supplementary material for: Longitudinal relationships between glycemic status and body mass index in a multiethnic study: evidence from observational and genetic epidemiology
Source: Sci Rep. 2016 Aug 2;6:30744. doi: 10.1038/srep30744 (PMC4969745; doi:10.1038/srep30744)
Supplement: Supplementary Information [file srep30744-s1.pdf]

Longitudinal relationships between glycemic status and body mass index in a multiethnic study: evidence from observational and genetic epidemiology

Adeola F. Ishola[1], Hertzel C. Gerstein[1,2,3], James C. Engert[4], Viswanathan Mohan[5], Rafael Diaz[6], Sonia S. Anand[1,2,3], David Meyre[1,2,7]

[1]Department of Clinical Epidemiology and Biostatistics, McMaster University, Hamilton, Ontario, Canada; [2]Population Health Research Institute, McMaster University and Hamilton Health Sciences, Hamilton General Hospital, Hamilton, Ontario, Canada; [3]Department of Medicine, McMaster University, Hamilton, Ontario, Canada; [4]Research Institute of the McGill University Health Centre, Montreal, QC, Canada; Departments of Medicine and Human Genetics, McGill University, Montreal, QC, Canada; [5]Madras Diabetes Research Foundation, Chennai, India; [6]ECLA—Academic Research Organization, Rosario, Argentina; [7]Department of Pathology and Molecular Medicine, McMaster University, Hamilton, Ontario, Canada.

Supplementary Table 1. Description of the 23 SNPs selected in EpiDREAM.

Gene	SNP	Risk Allele	Other Allele	Trait	Chromosome	Publication
TNNI3K	rs1514176	G	A	BMI	1	Speliotes, Nat Genet 2010
PCSK1	rs6235	C	G	Obesity	5	Benzinou, Nat Genet 2008
	rs6232	G	A			
CDKAL1	rs2206734	C	T	BMI	6	Okada, Nat Genet 2012
BDNF	rs6265	G	A	BMI	11	Thorleifsson, Nat Genet 2009
	rs1401635	C	G			
FTO	rs9939609	A	T	BMI	16	Frayling, Science 2007
	rs7203521	A	G			Thorleifsson Nat Genet 2009
NPC1	rs1805081	A	G	Obesity	18	Meyre Nat Genet 2009
TFAP2PB	rs2272903	G	A	BMI	6	Speliotes, Nat Genet 2010
NTRK2	rs1211166	A	G	BMI	9	Guo, Hum Mol Genet 2013
MAP2K5	rs997295	T	G	BMI	15	Speliotes, Nat Genet 2010
APOE	rs2075650	A	G	BMI	19	Guo, Hum Mol Genet 2013
GIPR	rs11671664	G	A	BMI	19	Speliotes, Nat Genet 2010
TAL1	rs2984618	T	G	BMI	1	Locke et al., Nat Genet 2015
LEPR	rs1011527	A	G	Obesity	1	Wheeler Nat Genet 2013
POMC	rs7605927	G	C	BMI	2	Graff Hum Mol Genet 2013
USP37	rs611203	G	A	BMI	2	Locke et al., Nat Genet 2015
ITIH4	rs2535633	G	C	BMI	3	Wen Hum Mol Genet 2014
NT5C2	rs3824755	C	G	BMI	10	Wen Hum Mol Genet 2014
TCF7L2	rs7903146	C	T	BMI	10	Locke et al., Nat Genet 2015
FANCL	rs12617233	C	T	BMI	2	Locke et al., Nat Genet 2015
KAT8	rs749767	A	G	BMI	16	Locke et al., Nat Genet 2015

Supplementary Table 2. Genotype distributions of 23 obesity SNPs in the EpiDREAM study.

Ethnicity	Major allele	Minor allele	Obesity risk allele	Genotype count			Obesity risk allele frequency	Genotype call rate	HWE *P*-value
**rs9939609– *FTO***									
				AA	AT	TT			
**South Asian**	T	A	A	323	1182	1251	0.332	100 %	0.088
**East Asian**				5	69	151	0.176	100 %	0.373
**European**				1698	4468	3214	0.419	100 %	0.035
**African**				293	640	314	0.491	100 %	0.345
**Latin American**				392	1440	1455	0.338	100 %	0.218
**Native North American**				32	166	301	0.230	100 %	0.165
**Total**				2743	7965	6686	0.387	100 %	N.A.
**rs7203521 – *FTO***									
				AA	GA	GG			
**South Asian**	A	G	A	521	1325	909	0.429	99.96%	0.328
**East Asian**				15	86	124	0.258	100 %	0.986
**European**				3538	4370	1472	0.610	100 %	0.045
**African**				503	569	175	0.632	100 %	0.489
**Latin American**				769	1548	969	0.469	99.97 %	0.002
**Native North American**				93	202	203	0.389	99.80 %	0.001
**Total**				5439	8100	3852	0.546	99.98 %	N.A.
**rs1514176 – *TNNI3K***									
				AA	GA	GG			
**South Asian**	A	G	G	565	1357	834	0.549	100 %	0.762
**East Asian**				35	70	120	0.689	100 %	$3.90x10^{-5}$
**European**				3173	4555	1652	0.419	100 %	0.804
**African**				137	551	559	0.669	100 %	0.944
**Latin American**				805	1564	918	0.517	100 %	0.010
**Native North American**				97	245	157	0.560	100 %	0.935
**Total**				4812	8342	4240	0.484	100 %	N.A.
**rs6265 – *BDNF***									
				AA	AG	GG			
**South Asian**	G	A	G	146	952	1658	0.774	100 %	0.540
**East Asian**				46	117	62	0.536	100 %	0.497
**European**				345	2807	6228	0.814	100 %	0.194
**African**				4	74	1169	0.967	100 %	0.018
**Latin American**				94	859	2334	0.841	100 %	0.166
**Native North American**				14	128	357	0.844	100 %	0.539
**Total**				649	4937	11808	0.821	100 %	N.A.
**rs1401635 – *BDNF***									
				CC	CG	GG			
**South Asian**	G	C	C	424	1255	1076	0.382	99.96 %	0.067
**East Asian**				4	28	193	0.080	100 %	0.020
**European**				826	3801	4752	0.291	99.99 %	0.094
**African**				77	478	692	0.253	100 %	0.645
**Latin American**				156	1119	2012	0.218	100 %	0.979
**Native North American**				28	168	303	0.224	100 %	0.462
**Total**				1515	6849	9028	0.284	99.99 %	N.A.
**rs1805081– *NPC1***									
				AA	GA	GG			
**South Asian**	A	G	A	1628	973	155	0.767	100 %	0.545
**East Asian**				133	77	15	0.762	100 %	0.402
**European**				3552	4385	1443	0.612	100 %	0.140
**African**				1083	158	6	0.92	100 %	0.928
**Latin American**				1588	1372	327	0.692	100 %	0.226
**Native North American**				224	231	44	0.680	100 %	0.151
**Total**				8208	7196	1990	0.679	100 %	N.A.
**rs6232– *PCSK1***									
				AA	GA	GG			
**South Asian**	A	G	G	2431	309	16	0.062	100 %	0.074
**East Asian**				223	2	0	0.004	100 %	0.947
**European**				8491	866	23	0.049	100 %	0.853

**African**				1229	18	0	0.007	100 %	0.797
**Latin American**				3099	185	3	0.029	100 %	0.889
**Native North American**				470	29	0	0.029	100 %	0.504
**Total**				15943	1409	42	0.043	100 %	N.A.
**rs6235 – *PCSK1***									
				CC	CG	GG			
**South Asian**	G	C	C	262	1096	1397	0.294	99.96 %	0.029
**East Asian**				20	93	112	0.296	100 %	0.912
**European**				684	3612	5083	0.265	99.99 %	0.225
**African**				27	329	891	0.154	100 %	0.600
**Latin American**				157	1084	2046	0.213	100 %	0.384
**Native North American**				27	188	284	0.242	100 %	0.568
**Total**				1177	6402	9813	0.252	99.99 %	N.A.
**rs2206734 – *CDKAL1***									
				CC	TC	TT			
**South Asian**	C	T	C	1623	974	159	0.766	100 %	0.421
**East Asian**				102	94	29	0.662	100 %	0.321
**European**				6015	3018	346	0.802	99.99 %	0.174
**African**				715	460	72	0.758	100 %	0.861
**Latin American**				2104	1047	136	0.799	100 %	0.689
**Native North American**				299	182	18	0.782	100 %	0.128
**Total**				10858	5775	760	0.790	99.99 %	N.A.
**rs2075650 – *APOE***									
				AA	GA	GG			
**South Asian**	A	G	A	2084	621	51	0.869	100 %	0.549
**East Asian**				173	47	5	0.873	100 %	0.402
**European**				6951	2248	181	0.861	100 %	0.962
**African**				956	278	13	0.878	100 %	0.144
**Latin American**				2593	647	47	0.887	100 %	0.361
**Native North American**				398	96	5	0.894	100 %	0.767
**Total**				13155	3937	302	0.870	100 %	N.A.
**rs2272903 – *TFAP2B***									
				AA	AG	GG			
**South Asian**	G	A	G	132	945	1679	0.781	100 %	0.948
**East Asian**				8	77	140	0.793	100 %	0.513
**European**				118	1778	7484	0.893	100 %	0.286
**African**				120	487	640	0.709	100 %	0.054
**Latin American**				72	783	2432	0.859	100 %	0.338
**Native North American**				4	99	396	0.893	100 %	0.417
**Total**				454	4169	12771	0.854	100 %	N.A.
**rs997295 – *MAP2K5***									
				GG	GT	TT			
**South Asian**	T	G	T	835	1336	585	0.455	100 %	0.239
**East Asian**				151	63	11	0.189	100 %	0.196
**European**				1597	4495	3288	0.590	100 %	0.363
**African**				261	616	370	0.544	100 %	0.876
**Latin American**				994	1584	708	0.456	99.97 %	0.102
**Native North American**				168	222	109	0.441	100 %	0.029
**Total**				4006	8316	5071	0.531	99.99 %	N.A.
**rs1211166 – *NTRK2***									
				AA	GA	GG			
**South Asian**	A	G	A	1436	1105	215	0.722	100 %	0.905
**East Asian**				151	63	11	0.811	100 %	0.196
**European**				6115	2898	365	0.806	99.98 %	0.351
**African**				567	540	139	0.671	99.92 %	0.542
**Latin American**				2114	1019	154	0.798	100 %	0.030
**Native North American**				378	111	10	0.869	100 %	0.582
**Total**				10761	5736	894	0.784	99.98 %	N.A.
**rs11671664 – *GIPR***									
				AA	AG	GG			
**South Asian**	G	A	G	33	521	2202	0.894	100 %	0.727
**East Asian**				41	89	95	0.620	100 %	0.016

**European**				122	1767	7488	0.892	99.97 %	0.126
**African**				15	256	976	0.885	100 %	0.696
**Latin American**				29	531	2726	0.910	99.97 %	0.577
**Native North American**				3	102	394	0.892	100 %	0.187
**Total**				243	3266	13881	0.892	99.98 %	N.A.
**rs2984618- *TAL1***									
				GG	GT	TT			
**South Asian**	G	T	T	554	1313	889	0.561	100 %	0.084
**East Asian**				3	19	203	0.944	100 %	0.003
**European**				3414	4445	1521	0.399	100 %	0.245
**African**				87	484	676	0.736	100 %	0.977
**Latin American**				766	1513	1008	0.537	100 %	$2.0x10^{-5}$
**Native North American**				115	248	136	0.521	100 %	0.924
**Total**				4939	8022	4433	0.485	100 %	N.A.
**rs1011527- *LEPR***									
				GG	GA	AA			
**South Asian**	G	A	A	1362	1144	250	0.299	100 %	0.660
**East Asian**				184	40	1	0.093	100 %	0.449
**European**				8038	1284	56	0.074	99.98 %	0.544
**African**				928	293	26	0.138	100 %	0.612
**Latin American**				2845	428	13	0.069	99.97 %	0.467
**Native North American**				450	47	2	0.051	100 %	0.520
**Total**				13807	3236	348	0.113	99.98 %	N.A.
**rs7605927- *POMC***									
				GG	CG	CC			
**South Asian**	C	G	C	586	1364	794	0.536	99.56 %	0.996
**East Asian**				73	113	39	0.424	100 %	0.675
**European**				577	3386	5413	0.758	99.96 %	0.123
**African**				263	578	406	0.557	100 %	0.032
**Latin American**				508	1491	1282	0.617	99.82 %	0.031
**Native North American**				77	215	207	0.630	100 %	0.092
**Total**				2084	7147	8141	0.673	99.87 %	N.A.
**rs611203- *USP37***									
				AA	GA	GG			
**South Asian**	A	G	G	1351	1101	303	0.310	99.96 %	0.001
**East Asian**				149	66	10	0.191	100 %	0.442
**European**				3264	4517	1599	0.411	100 %	0.591
**African**				452	616	179	0.391	100 %	0.183
**Latin American**				1094	1630	563	0.420	100 %	0.292
**Native North American**				218	219	62	0.344	100 %	0.544
**Total**				6528	8149	2716	0.390	99.99 %	N.A.
**rs2535633- *ITIH4***									
				GG	CG	CC			
**South Asian**	G	C	C	456	1287	1013	0.399	100 %	0.168
**East Asian**				27	111	87	0.367	100 %	0.351
**European**				1565	4503	3309	0.407	99.97 %	0.618
**African**				584	538	125	0.684	100 %	0.946
**Latin American**				774	1618	894	0.482	99.97 %	0.426
**Native North American**				123	245	131	0.492	100 %	0.691
**Total**				3529	8302	5559	0.442	99.98 %	N.A.
**rs3824755- *NT5C2***									
				GG	CG	CC			
**South Asian**	G	C	C	178	961	1617	0.761	100 %	0.030
**East Asian**				15	100	110	0.711	100 %	0.220
**European**				103	1671	7606	0.900	100 %	0.297
**African**				45	395	807	0.806	100 %	0.696
**Latin American**				93	903	2291	0.834	100 %	0.723
**Native North American**				8	122	369	0.862	100 %	0.562
**Total**				442	4152	12800	0.855	100 %	N.A.
**rs7903146- *TCFL72***									
				TT	CT	CC			
**South Asian**	C	T	C	272	1170	1314	0.689	100 %	0.624

**East Asian**				2	22	201	0.942	100 %	0.126
**European**				956	3903	4521	0.690	100 %	0.01
**African**				112	520	615	0.702	100 %	0.890
**Latin American**				280	1304	1703	0.716	100 %	0.177
**Native North American**				10	145	344	0.835	100 %	0.238
**Total**				1632	7064	8698	0.703	100 %	N.A.
**rs126172333- *FANCL***									
				TT	TC	CC			
**South Asian**	G	A	G	352	1301	1103	0.636	100 %	0.297
**East Asian**				67	119	39	0.438	100 %	0.264
**European**				1537	4480	3363	0.597	100%	0.489
**African**				152	579	516	0.646	100 %	0.593
**Latin American**				638	1583	1066	0.565	100 %	0.247
**Native North American**				76	247	176	0.600	100 %	0.483
**Total**				2822	8309	6263	0.599	100 %	N.A.
**rs749767- *KAT8***									
				GG	GA	AA			
**South Asian**	A	G	A	52	620	2082	0.868	99.93 %	0.461
**East Asian**				154	60	11	0.182	100 %	0.114
**European**				1437	4477	3466	0.608	100 %	0.889
**African**				111	496	640	0.712	100 %	0.291
**Latin American**				715	1559	1012	0.545	99.97 %	0.013
**Native North American**				109	232	158	0.549	100 %	0.172
**Total**				2578	7444	7369	0.638	99.98 %	N.A.

Supplementary Table 3. Proportion of subjects with NGT, IFG/IGT and T2D within each ethnic groups at baseline.

Ethnicity	***NGT***	***IFG/IGT***	***T2D***	***All***
South Asian	1566 (56.5)	756 (27.4 %)	4454 (16.1 %)	2756 (100 %)
East Asian	74 (32.9 %)	100 (44.4 %)	51 (22.7 %)	225 (100 %)
European	3671 (39.1 %)	4355 (46.5 %)	1354 (14.4 %)	9380 (100 %)
African	465 (37.3 %)	557 (44.8 %)	225 (18.0 %)	1247 (100 %)
Latino American	1460 (44.4 %)	1402 (42.7 %)	425 (12.9 %)	3287 (100 %)
Native North American	221 (44.3 %)	219 (43.9 %)	59 (11.8 %)	499 (100 %)

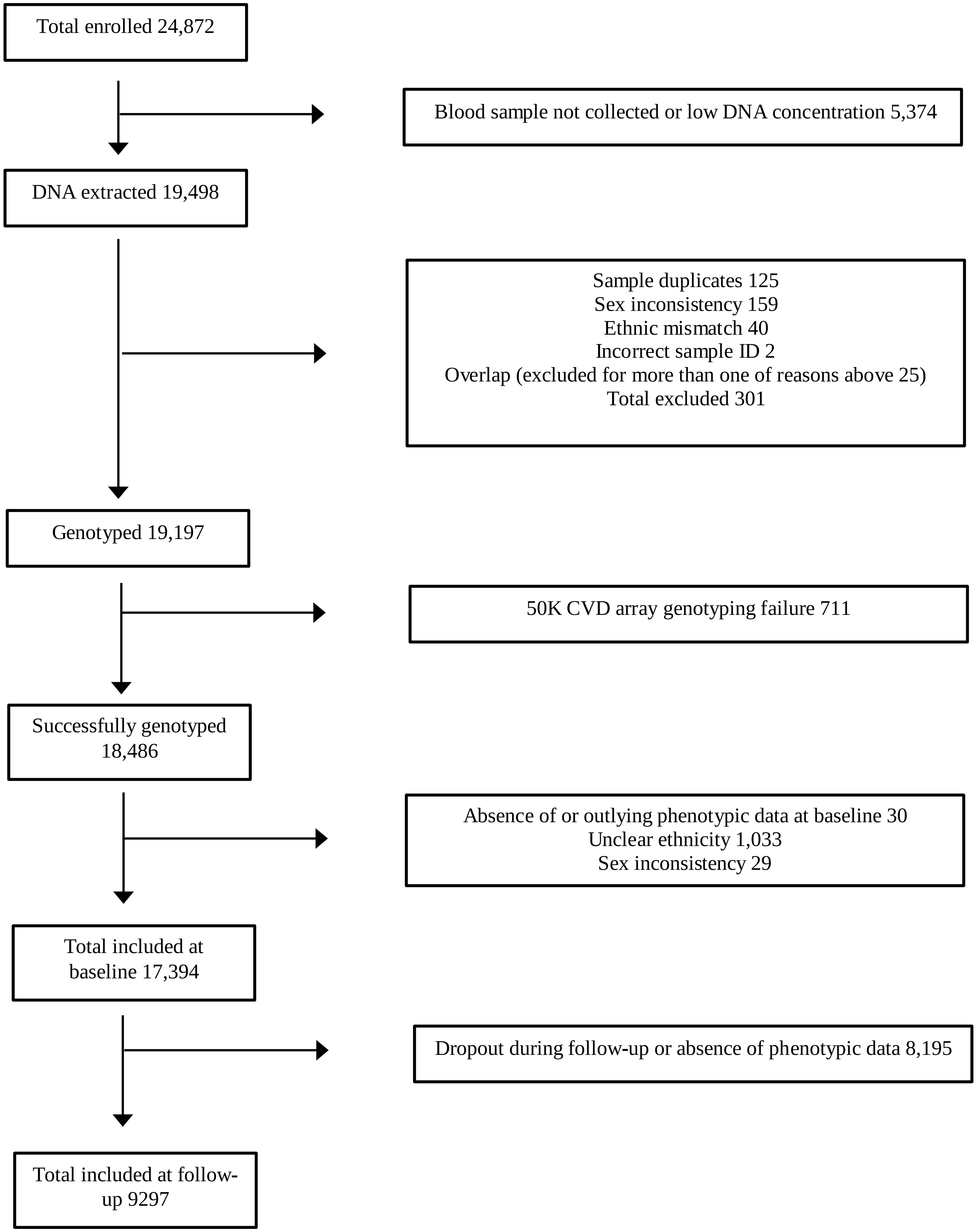

Supplementary Figure 1. EpiDREAM study selection process overview.

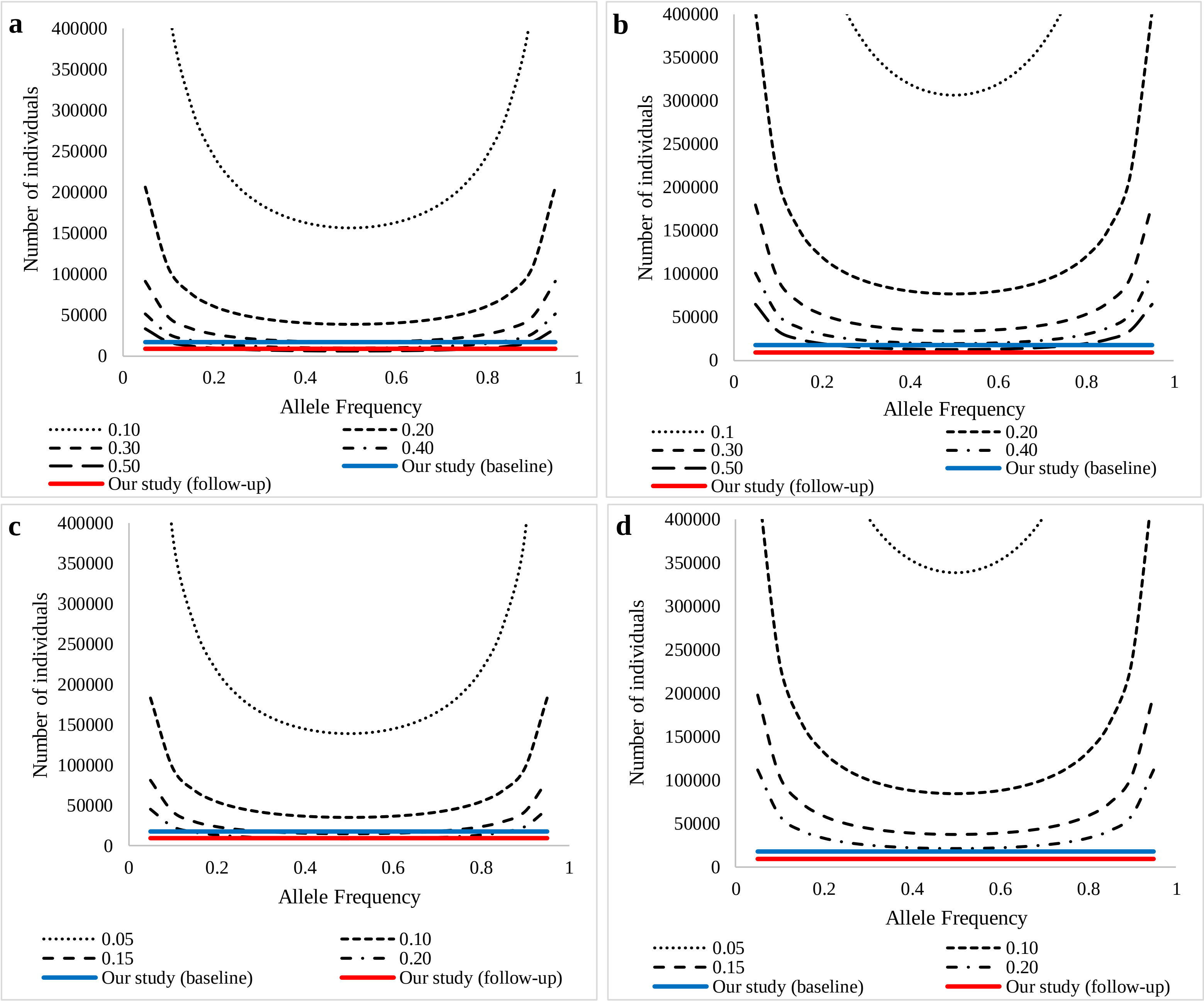

Supplementary Figure 2. Number of individuals required for a statistical power of 80% based on obesity risk allele frequency and beta effect values. a) Main effect analysis using a *P*-value of 0.05, unadjusted for multiple testing; b) Main effect analysis after adjusting for multiple testing (adjusted *P*-value $=2.1\text{x}10^{-3}$) with the Bonferroni correction method; c) Gene-environment interaction effect analysis using a *P*-value of 0.05, unadjusted for multiple testing; d) Gene environment interaction effect after adjusting for multiple testing (adjusted *P*-value = $4.2\ \text{x}10^{-4}$ with the Bonferroni correction method).
